# Determinants of the risk of dying of HIV/AIDS in a rural South African community over the period of the decentralised roll-out of antiretroviral therapy: a longitudinal study

**DOI:** 10.3402/gha.v7.24826

**Published:** 2014-11-20

**Authors:** Paul Mee, Mark A. Collinson, Sangeetha Madhavan, Chodziwadziwa Kabudula, Francesc Xavier Gómez-Olivé, Kathleen Kahn, Stephen M. Tollman, James Hargreaves, Peter Byass

**Affiliations:** 1 Medical Research Council/Wits University Rural Public Health and Health Transitions Research Unit (Agincourt), School of Public Health, Faculty of Health Sciences, University of the Witwatersrand, Johannesburg, South Africa; 2 Umeå Centre for Global Health Research, Department of Public Health and Clinical Medicine, Umeå University, Umeå, Sweden; 3 Department of Global Health and Development, Faculty of Public Health and Policy, London School of Hygiene and Tropical Medicine, London, UK; 4 International Network for the Demographic Evaluation of Populations and Their Health (INDEPTH) Network, Accra, Ghana; 5 Department of African-American Studies, University of Maryland-College Park, College Park, MD, USA; 6 Department of Social and Environmental Health Research, Faculty of Public Health and Policy, London School of Hygiene and Tropical Medicine, London, UK; 7 WHO Collaborating Centre for Verbal Autopsy, Umeå Centre for Global Health Research, Department of Public Health and Clinical Medicine, Umeå University, Umeå, Sweden

**Keywords:** HIV, mortality, determinants, global health, population health, healthcare access, South Africa

## Abstract

**Background:**

Antiretroviral treatment (ART) has significantly reduced HIV mortality in South Africa. The benefits have not been experienced by all groups. Here we investigate the factors associated with these inequities.

**Design:**

This study was located in a rural South African setting and used data collected from 2007 to 2010, the period when decentralised ART became available. Approximately one-third of the population were of Mozambican origin. There was a pattern of repeated circular migration between urban areas and this community. Survival analysis models were developed to identify demographic, socioeconomic, and spatial risk factors for HIV mortality.

**Results:**

Among the study population of 105,149 individuals, there were 2,890 deaths. The HIV/TB mortality rate decreased by 27% between 2007–2008 and 2009–2010. For other causes of death, the reduction was 10%. Bivariate analysis found that the HIV/TB mortality risk was lower for: those living within 5 km of the Bhubezi Community Health Centre; women; young adults; in-migrants with a longer period of residence; permanent residents; and members of households owning motorised transport, holding higher socioeconomic positions, and with higher levels of education. Multivariate modelling showed, in addition, that those with South Africa as their country of origin had an increased risk of HIV/TB mortality compared to those with Mozambican origins. For males, those of South African origin, and recent in-migrants, the risk of death associated with HIV/TB was significantly greater than that due to other causes.

**Conclusions:**

In this community, a combination of factors was associated with an increased risk of dying of HIV/TB over the period of the roll-out of ART. There is evidence for the presence of barriers to successful treatment for particular sub-groups in the population, which must be addressed if the recent improvements in population-level mortality are to be maintained.

In South Africa, in 2013, there were 6.3 million people living with HIV and just over 340,000 new infections. Among those aged 15–49, the HIV prevalence was 19.1%, and 2.6 million people were receiving antiretroviral treatment (ART) (1). The roll-out of ART in South Africa and other sub-Saharan African countries has led to significant reductions in population-level mortality (2–4). There were estimated to have been 200,000 HIV-related deaths in South Africa in 2013, down from a peak of 410,000 in 2010; the decrease being mainly due to the roll-out of ART in the public health sector (5). Studies have shown that communities in close proximity may have micro-epidemics of HIV disease with quite different characteristics (6–10). Identification of groups not benefitting from general improvements in HIV mortality could enable targeted local interventions (11, 12). The factors defining these sub-groups of individuals may be geographic, related to the physical barriers to accessing healthcare (13–15), environmental, or associated with socioeconomic or demographic factors. Research in the site of the present study identified local geographic variations in both HIV mortality rates and the changes in those rates over the period that a decentralised programme of ART was implemented (16). This indicated that the benefits of ART have not been equally experienced by all groups.

In South Africa and surrounding countries, patterns of migration, both within the country and across national boundaries, have had a significant effect on the social and ethnic structure of the society. There have been various waves of mass in-migration in recent decades. In the region of South Africa where this study was located, the in-migrants were predominantly Mozambicans fleeing from the civil war which ended in the early 1990s (17). In South Africa, a vast pool of labour was required to support the gold mining industry which developed rapidly from the early 20th century. To meet this need, men were brought in from rural areas, whilst their families remained in the rural communities. Apartheid era legislation prevented these workers from permanently settling with their families in the urban areas. A pattern of temporary or circular migration subsequently developed between urban areas, where the men spent most of their time, and rural communities. This pattern continues to the present day (18–20).

Associations between barriers to clinic access, ART uptake, and mortality have been identified in sub-Saharan Africa. A study in a rural area of KwaZulu Natal, South Africa, between 2004 and 2008 found that ART uptake was strongly negatively associated with the distance individuals lived from the nearest primary healthcare facility (21). Studies in rural areas of Malawi (22) and Uganda (23) in 2006 found that ART acceptance was negatively associated with cost of public transport to the hospital providing treatment (22). An analysis of mortality trends in the site of the current study on data collected from 1993 to 2010 found a significant association between the straight line distance to a clinic and adult mortality in bivariate models, although the significance was lost after multivariate adjustment (24). Other studies have identified risk factors for all-cause and cause-specific mortality in adults and children in the same area (24–26).

Migration patterns have been shown to have a considerable influence on mortality risks, particularly the risks associated with HIV mortality, in this setting. A significant increase in HIV and tuberculosis (TB) related mortality has been reported in the first 5 years after migrants return home compared with those who had not migrated (27–29). The relationship between temporary migration and mortality is complex, with the risks varying for different types of individuals (30). The increased risk of dying of communicable diseases, principally HIV/AIDS, among temporary migrants decreased significantly in the area of the current study between 2000 and 2011 (31). Others have described similar phenomena elsewhere in the region (32–34).

Risk factors associated with HIV infection may differ from those associated with HIV-related mortality. For example, those of higher socioeconomic position (SEP) may be at a higher risk of infection, at least in the early stages of a local epidemic (35), whilst at the same time having the means to overcome financial barriers to accessing treatment (36). However, a comparison of determinants of the risk of HIV infection from other studies with determinants of mortality may provide useful insights.

A meta-analysis concluded that it was not wealth *per se*, but rather the level of socioeconomic inequity which was the most important risk factor for HIV infection (37). It has been suggested (35) that the relationship may depend on the stage of the epidemic, with wealthier individuals preferentially benefitting from the availability of new treatments. Another meta-analysis has shown a similar time-dependent relationship between education and the risk of HIV infection (38), with more highly educated individuals at higher risk of infection earlier in the epidemic and their risk decreasing as the epidemic progresses.

In this study, we used a survival analysis approach developing bivariate and multivariate Poisson regression models to assess whether variables were associated with the risk of HIV-related, non-HIV, and all-cause mortality. We investigated the extent to which the associations were age-specific and whether they changed over the period of the analysis. These models were used to describe the associations between the risk of death and determinants describing: the distance to healthcare facilities, the cost of travelling to these facilities and other socio-economic and demographic factors, over the period that a decentralised provision of ART was established in a rural community in South Africa. A study including data collected up until the end of December 2010 indicated that for those living in the study area, being Mozambican was not a risk factor for mortality (24); this contrasted with studies using earlier data which indicated that those of Mozambican origin had a higher risk of death (39, 40). In order to better understand this association, country of origin was included as a variable in the multivariate models.

## Present investigation

### Methods

#### Setting and data collection

**Fig. 1:**
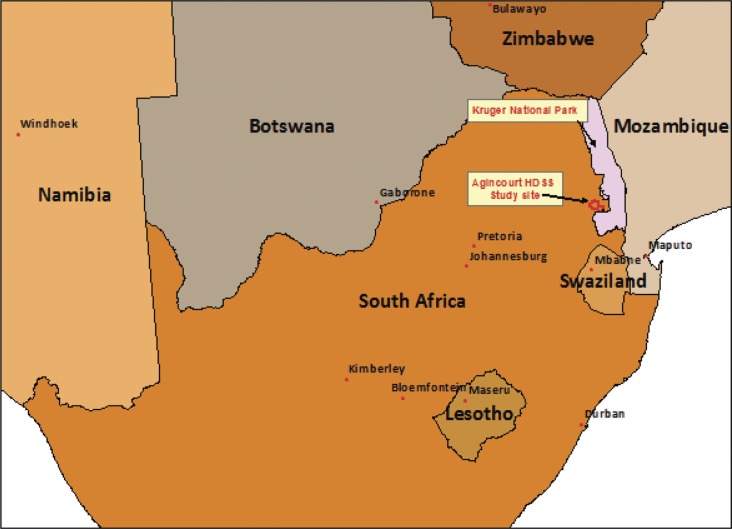
The location of the Agincourt HDSS in the South Africa and the surrounding region.

This study used data collected in the Agincourt health and socio-demographic survey site (HDSS). The site is located in the Bushbuckridge sub-district of Ehlanzeni municipality of Mpumalanga province in South Africa. It is a predominantly rural area with neighbouring peri-urban communities in close proximity (41). The area is 70 km west of the border with Mozambique, the two being separated by the Kruger National Park (Fig. 1). There are strong ethnic and cultural links between those in present day Mozambique and this region (17). In more recent years, many migrants fleeing from the Mozambican civil war settled in the site. Approximately one-third of the population is of Mozambican origin.

The Agincourt HDSS was established in 1992. By 2010, the end of the period of this study, the population under surveillance was approximately 90,000 individuals in 16,000 households. Visits were made annually to each household in the study site in order to update data on vital events occurring to all residents in the household. Refusal rates were very low (less than five households per year). When a death was reported, a verbal autopsy (VA) questionnaire was administered to collect information on the symptoms of the preceding illness and the events leading up to the death (42). Fieldworkers were trained to identify the informant with the greatest knowledge of the household members who was then interviewed to complete the annual update. VA questionnaires were completed by interviewing the main caregiver of the deceased during their final illness. The types of data collected in the Agincourt HDSS database and the underlying data model have been described previously (41, 43).

ART programmes commenced at two secondary hospitals serving the Agincourt HDSS site in 2004 and 2005. A community health centre (CHC), Bhubezi, set up as a public–private partnership, was opened in the study site in 2007 in order to provide general healthcare with a particular emphasis on providing treatment and testing for those with HIV. As part of an initiative to decentralise public sector health services, one clinic within the study site and one on periphery began to provide ART services in 2008 but until 2010 there were only small numbers of patients, approximately 100 per year, obtaining ART at the latter two clinics. In comparison, close to 1,600 patients had commenced ART at Bhubezi CHC by February 2009. The total number on ART is likely to be higher than this as labour migrants who remain linked to the local household may be receiving treatment close to their place of work.

An HIV prevalence survey of adults conducted in the study site between August 2010 and May 2011 showed an overall HIV prevalence of 19.4% with a large sex gap (10.6% for males and 23.9% for females); peak rates of just over 45% were seen among males and females aged 35–39. This study also found that South Africans had a higher probability of being HIV positive than former Mozambican refugees resident in the study site (44).

#### Defining the study population

The target population was defined as the total number of individuals under surveillance at the midpoint of the analysis, 1 January 2009, excluding residents of two villages added to the study site in 2009. For the cause-specific mortality analyses, individuals with an unknown cause of death (CoD) or those who were missing data for determinants included in the regression models were excluded from the study population.

#### CoD assignment

The InterVA-4 model was used to assign CoD using a Bayesian probabilistic approach to assess the likelihood of each possible cause based on the presence of particular signs and symptoms (45). The CoDs were categorised according to the WHO 2012 VA standard (46). The input variables for the model were derived by matching variables in the Agincourt VA questionnaire (47) or identifying specific text strings in the narrative fields. In the latter case, the Jaro–Winkler algorithm (48) with a cut-off score of 0.9 was used, which enabled misspelt words to be identified. A recent study pooling data from six sites in sub-Saharan Africa indicated that the rate ratio of those HIV positive to HIV negative among those dying of pulmonary TB was 63.9 (49). Others have reported a high level of HIV/TB co-morbidity in this region (50). Thus, the vast majority of those dying of pulmonary TB are likely to be HIV co-infected, and the underlying HIV infection is likely to have been a contributory factor in their death. Hence, in this study, deaths due to HIV disease or pulmonary TB were combined in a single category HIV/TB.

All other deaths for which a cause was assigned were categorised as non-HIV/TB.

#### Derivation of variables

Clinic access To calculate road distances to Bhubezi CHC, a network model of the major roads within the study site was created using the ArcGIS software Network Analyst module (51). Where geographical coordinates for a dwelling were not available the location was assigned to the geometric centre of the village. To measure public transport costs to Bhubezi CHC a survey of private taxi associations was carried out in June 2010. Data on household transport ownership were derived from one component of the household asset survey.

Socioeconomic position and education In the Agincourt HDSS, household asset survey information was collected on 34 ordinal variables covering features such as the type of household building materials used, access to water and fuel, and the ownership of appliances, livestock, and transport. From these data five sub-indicators were constructed within which each asset variable was weighted equally. The sub-indicators were combined and standardised leading to an absolute SEP score. Using this, households were ranked into one of five quintiles allowing the relative asset status of households at a particular point in time to be compared (30, 52). A full listing of the component variables used to create the asset index is included in Supplementary Table 6. If no data was available for a particular household in 2009, the SEP quintile from the next most recent preceding year for which it was available was used. The SEP quintiles were recalculated omitting the transport ownership variable in order to assess whether this affected the associations with mortality risk. In the 2009 education survey, data were collected on the number of years of education successfully completed for each household member. If an individual successfully progressed each year, it would take 12 years of school attendance to complete primary and secondary education. The mean number of years of education completed for all those aged over 18 in each household was calculated. This variable describes the overall educational level in the home. A comparison was made of strata-specific mortality rates between the average adult household education variable and the individual level of education among those over 18 years of age as used in previous studies.

Residence status Individuals were defined as permanent residents if they dwelt in the study site for more than 6 months of the year and as temporary migrants otherwise.

Migration For those who had migrated into a household, the place of origin and the time between their arrival and exit from the analysis were included. Place of origin was divided into four categories; Agincourt area (those who had arrived from another village in the survey site), Bushbuckridge (the municipality of which Agincourt is a part, excluding migrants from the survey site), Gauteng and other urban areas (Gauteng province includes Johannesburg and Pretoria), and Mozambique. No cases of international migration from countries other than Mozambique were recorded in the Agincourt HDSS. Individuals defined as being of Mozambican origin include those born in Mozambique and the children of fathers born in Mozambique. Other variables were extracted directly from the Agincourt HDSS database.

### Analytical approach

Data were analysed using Stata, version 10.0 SE (53). Chi-square tests were used for categorical variables to check for collinearity before using these variables in the model.

In order to assess the influence of missing data on the outcome, an analysis was made of whether the mean values for continuous variables or the distribution of categorical variables differed between the study populations and the excluded individuals. A two-sampled *t*-test was used to test for differences in the mean values (54), and the Pearson's chi-square test (55) was used to assess whether the distributions of categorical variables differed. The results are shown in Supplementary Tables 8 and 9.

Person time was accrued from either the beginning of the analysis (1 January 2007) or the date of in-migration, whichever was later, until right censoring at either an outmigration, death, or the end of analytical period (31 December 2010). Poisson regression models were developed in which individuals were clustered by household in order to include household level random effects (56). To assess the population-level impact of the local ART roll-out we divided the analysis into two time periods 2007 to 2008 and 2009 to 2010, representing the periods immediately prior and subsequent to the point at which the impact of the ART provision from Bhubezi CHC on HIV/TB-related mortality in the study site would be expected to be evident.

Initially, a series of bivariate models were developed including each individual explanatory variable in turn and a failure due to an HIV/TB death. Incident rate ratios (IRR), the ratio between the mortality rates for individuals with a particular level of a categorical variable and individuals in the baseline category (57), *p*-values, and 95% confidence intervals were derived. Subsequently, those variables which showed an independent association with the outcome (*p<*0.10) for at least one level of the categorical variables, or were of interest based on *a priori* hypotheses, were included in the multivariate regression models. This was repeated for failures due to non-HIV/TB death. Interaction terms were added individually to the multivariate models to test whether the associations differed according the age, SEP, or time period. Interaction terms were retained if they improved the predictive ability of the model.

In order to test whether differences in associations between the two causes of death were statistically significant, a further multivariate model including a dichotomous variable defining the death type was developed in which the outcome was mortality due to any known cause. Interaction terms between CoD and other variables were added to assess whether the associations with the outcome differed by CoD.

## Results

In total, 113,253 individuals were resident in the villages in the study area between 1 January 2007 and 31 December 2010. They contributed 335,392 person years of observation (PYO) and had a crude mortality rate of 10.8 deaths/1,000 PYO (Supplementary Table 1). This is consistent with data showing a death rate of 11.5 deaths/1,000 PYO in 2010 for Mpumalanga province in which the study site is located (58). After exclusions due to missing data for covariates or unknown causes of death, there remained 105,149 individuals among whom there were 2,890 deaths. They contributed 320,945 PYO and had a crude mortality rate of 9.0 deaths/1,000 PYO. Lower mortality rates for the study population than the target population were seen in all age strata, the effect being particularly large for those in the 0–4 year category. There were 735 deaths in the excluded individuals and they contributed 14,447 PYO; the death rate in this group was 50.9 deaths/1,000 PYO. There were a relatively high proportion of neonates (0–1 year) who were excluded (11.3% of all excluded and 25.2% of all neonatal deaths) (Supplementary Table 7). This was due mainly to there being a high percentage of indeterminate causes of data assigned to those dying in the first year of life. This had a disproportionate effect on the overall mortality rate due to the small amount of person time these individuals contributed.

We compared mean values or distributions of variables describing the characteristics of individuals and their households between the study population and those excluded (Supplementary Tables 8 and 9). Significant differences were seen in all cases other than for the mean value of the average number of years of education completed for the household.

The predominant causes of death in the study population were HIV disease (25.1%), pulmonary TB (19.2%), and acute respiratory infections including pneumonia (17.0%) (Supplementary Table 2).

Among the study population, 55,938 (53.2%) were female, 70,812 (67.3%) were South Africans, and 79,695 (77.0%) were permanent residents. Males were, on average, younger than females (23.32 vs. 25.93 years), had higher mean household SEP (average quintile value 3.18 vs. 3.14), and had higher levels of mean household education (7.73 vs. 7.68 years) (Table 1).

**Table 1:** Descriptive analysis of study population broken down by sex

Variable	Category	Category total (%)	Sex		*p* a

			Female *N* (%/95% CI)	Male *N* (%/95% CI)	
Country of origin	Mozambique	**34,337 (32.66)**	18,032 (52.51)	16,305 (47.49)	0.002
	South Africa	**70,812 (67.34)**	37,906 (53.53)	32,906 (46.47)	
Residence status	Temporary	**25,330 (23.00)**	9,596 (39.68)	14,587 (60.32)	<0.001
	Permanent	**79,695 (77.00)**	46,342 (57.24)	34,624 (42.76)	
Mean age^b^	–	–	25.93 (25.76–26.10)	23.32 (23.15–23.48)	<0.001
Mean socioeconomic position	–	–	3.14 (3.13–3.16)	3.18 (3.17–3.19)	<0.001
Mean household education (years)	–	–	7.68 (7.66–7.71)	7.73 (7.71–7.76)	<0.001
	Total	105,149	55,938 (53.20)	49,211 (46.80)	<0.001

a *P* values calculated using chi-squared for categorical variables and *t*-test for continuous variables.

b Includes only individuals resident in the study site on 1st January 2009, the mid-point of this analysis.

### Bivariate and multivariate analyses of the risk of HIV/TB death

There was a decrease in the mortality rates between 2007–2008 and 2009–2010 for those dying of HIV and TB (IRR=0.72, 95% CI: 0.65–0.81) (Table 2).

**Table 2:** Bivariate and multivariate analyses of risk factors for deaths due to HIV/TB

Variable	Category	Deaths/PYO	Bivariate analysis			Multivariate analysis^a^	
	
			Mortality rate deaths/1,000 PYO	Rate ratio	95% Confidence interval (*p* value)	Rate ratio	95% Confidence interval (*p* value)
Time period	2007–2008	718/154,449	4.65				
	2009–2010	560/166,496	3.36	0.72	0.65–0.81 (<0.001)	0.70	0.63–0.79 (<0.001)
Sex	Female	611/166,715	3.66				
	Male	667/154,229	4.32	1.18	1.06–1.32 (0.003)	1.28	1.14–1.44 (<0.001)
Age group	15–49	780/170,521	4.57				
	<5	87/39,035	2.23	0.49	0.39–0.61 (<0.001)	0.49	0.39–0.62 (<0.001)
	5–14	31/72,418	0.43	0.09	0.06–0.14 (<0.001)	0.09	0.06–0.14 (<0.001)
	50–65	220/23,793	9.25	2.02	1.74–2.34 (<0.001)	2.07	1.78–2.41 (<0.001)
	>65	160/15,177	10.54	2.30	1.94–2.73 (<0.001)	2.25	1.87–2.72 (<0.001)
Country of origin	Mozambican	413/105,023	3.93				
	South African	865/215,922	4.01	1.02	0.90–1.15 (0.765)	1.20	1.05–1.38 (0.009)
Residence status	Temporary	405/74,346	5.45				
	Permanent	873/246,599	3.54	0.65	0.58–0.73 (<0.001)	0.80	0.70–0.90 (<0.001)
Time since in-migration	Non-migrant	680/175,266	3.88				
(years)	0–3	166/34,141	4.86	1.25	1.05–1.49 (0.011)	1.50	1.25–1.80 (<0.001)
	4–6	132/35,076	3.76	0.97	0.80–1.18 (0.757)	1.09	0.90–1.33 (0.377)
	7–9	111/23,892	4.65	1.20	0.98–1.47 (0.085)	1.24	1.01–1.53 (0.042)
	10–12	80/21,316	3.75	0.97	0.76–1.23 (0.783)	0.90	0.70–1.14 (0.367)
	>12	109/31,254	3.49	0.90	0.73–1.10 (0.303)	0.70	0.57–0.86 (0.001)
Place of origin for	Non-migrant	680/175,266	3.88				
in-migrant	Agincourt Area	468/175,266	4.09	1.05	0.93–1.19 (0.396)		
	Bushbuckridge	72/114,488	4.02	1.04	0.81–1.33 (0.785)		
	Gauteng/Urban	52/17,918	4.49	1.16	0.87–1.53 (0.312)		
	Mozambique	6/11,584	3.55	0.92	0.41–2.04 (0.83)		
Taxi fare (rands)	<17	302/1,688	3.81				
	17–36	639/79,296	4.04	1.06	0.92–1.22 (0.401)		
	>36	337/79,296	4.04	1.06	0.91–1.24 (0.467)		
Distance by road from	0–5	223/158,155	3.50				
the Bhubezi CHC (km)	>5	1055/83,493	4.10	1.17	1.01–1.36 (0.038)	1.17	1.01–1.37 (0.042)
SEP quintiles	1		6.05				
	2	262/257,298	4.22	0.70	0.59–0.82 (<0.001)	0.77	0.65–0.91 (0.002)
	3	264/45,478	3.90	0.64	0.54–0.77 (<0.001)	0.75	0.62–0.90 (0.002)
	4	246/62,150	3.54	0.59	0.49–0.70 (<0.001)	0.72	0.60–0.87 (0.001)
	5	231/67,732	3.04	0.50	0.42–0.60 (<0.001)	0.69	0.56–0.85 (<0.001)
Transport ownership	No household transport	1,025/69,527	4.51				
by household	Non-motorised transport	79/76,057	3.27	0.73	0.57–0.92 (0.009)	0.80	0.62–1.02 (0.068)
	Motorised transport (car or motorbike)	174/227,473	2.51	0.56	0.47–0.66 (<0.001)	0.68	0.57–0.81 (<0.001)
Mean level of adult	0–5	341/24,172	5.59				
household education	6–7	364/69,300	4.53	0.81	0.70–0.94 (0.006)	0.86	0.73–1.01 (0.063)
(years)	8–9	355/60,953	3.75	0.67	0.57–0.78 (<0.001)	0.71	0.60–0.85 (<0.001)
	10–11	171/80,363	2.70	0.48	0.40–0.58 (<0.001)	0.54	0.44–0.66 (<0.001)
	>11	47/94,700	2.17	0.39	0.29–0.53 (<0.001)	0.48	0.35–0.67 (<0.001)

a The multivariate model included variables which were showed a significant association in at least one strata in the bivariate model or were included to test *a priori* hypotheses about their significance. Time period, sex, age, country of origin, place of origin, residence status, time since in-migration, road distance from Bhubezi Community Health Centre (CHC), SEP, transport ownership, and average level of household education were included.

In the bivariate analysis, there was evidence that those living further than 5 km from the Bhubezi CHC had a higher risk of dying of HIV/TB than those living within walking distance (less than 5 km) (IRR=1.17, 95% CI: 1.01–1.36). Rates were lower for those living in households which owned a motorised form of transport than for those with no transport (IRR 0.56, 95% CI: 0.47–0.66). There were no significant associations between taxi fare to Bhubezi CHC and the risk of HIV/TB death. Household SEP and the mean level of adult household education were both strongly associated with the risk of dying of HIV/TB with those in all strata over the baseline at a decreased risk. Males had significantly higher rates than females (IRR 1.18, 95% CI: 1.06–1.32). The rate for children younger than 5 years and aged 5–14 years was significantly lower than that for those aged 15–49. Whilst for the older age groups the rate was more than double that for those aged 15–49. Those of Mozambican origin had virtually identical HIV/TB mortality rates to South Africans. Permanent residents had lower rates than temporary migrants (IRR 0.65, 95% CI: 0.58–0.73). Those in-migrating in the 3 years prior to the end of follow-up had higher HIV/TB mortality rates than the non-migrants (IRR 1.25, 95% CI: 1.05–1.49).

A comparison among those aged 18 and over at the start of the study period between the average adult household education variable as used in this analysis and the individual level of education showed that, in each case, higher education levels led to a decreased risk of HIV mortality (Supplementary Table 3). In the case of individual education, however, a decrease in risk was only seen for those who had successfully completed 10 or more years of schooling and there was evidence that those with 6–7 years of education were at a higher risk than those with 0–5 years. For the average household education variable, the risk of HIV mortality was seen at all levels above the baseline.

In the multivariate analysis in order to investigate *a priori* hypotheses about health-seeking behaviour of individuals from different countries of origin, a variable describing this characteristic was included, despite it not being identified as a risk factor in the bivariate analysis. South Africans were seen to have a higher risk of HIV/TB death than those of Mozambican origin (IRR 1.20, 95% CI: 1.05–1.38). The decreased risk associated with transport ownership and living less than 5 km of Bhubezi CHC remained unchanged. The associations with sex, time period, age, SEP, and education were very similar to those seen in the bivariate analysis. Alternative models were created using an SEP variable computed without the transport ownership component. This made no significant difference to the associations with mortality risk. The original SEP variable was thus retained to enable comparison with previous studies.

There was evidence for an interaction between the time period and ownership of transport by a household. The HIV/TB mortality rate ratio for households owning transport compared to those without was 1.33 times higher in 2009–2010 than in 2007–2008 (95% CI: 1.00–1.77) (Supplementary Table 4). The protective effects of higher levels of mean adult household education (>7 years vs. 7 years or less, IRR=0.70 95% CI: 0.47–1.045) and household transport ownership (transport ownership vs. no transport ownership, IRR=0.60 95% CI: 0.34–1.06) were lower in those younger than 15 than those aged 15 and above (Supplementary Table 5). There also was no evidence for an interaction between transport ownership and the distance an individual lived from Bhubezi CHC.

### Bivariate and multivariate analyses of the risk of non-HIV/TB death

There was evidence for a decrease in the mortality rates between 2007–2008 and 2009–2010 for causes of death other than HIV and TB (IRR=0.90 95% CI: 0.81–0.99); the decrease was less than that for HIV/TB deaths (Table 3).

**Table 3:** Bivariate and multivariate analyses of risk factors for deaths due to causes other than HIV/TB

Variable	Category	Deaths/PYO	Bivariate analysis			Multivariate analysis^a^	
	
			Mortality rate deaths/1,000 PYO	Rate ratio	95% Confidence interval (*p* value)	Rate ratio	95% Confidence interval (*p* value)
Time period	2007–2008	820/154,449	5.31				
	2009–2010	792/166,496	4.76	0.90	0.81–0.99 (0.027)	0.87	0.79–0.97 (0.008)
Sex	Female	757/166,715	4.54				
	Male	855/154,229	5.54	1.22	1.11–1.35 (<0.001)	1.53	1.38–1.69 (<0.001)
Age group	15–49	497/170,521	2.91				
	<5	302/39,035	7.74	2.65	2.29–3.08 (<0.001)	2.37	2.04–2.75 (<0.001)
	5–14	46/72,418	0.64	0.22	0.16–0.29 (<0.001)	0.20	0.15–0.28 (<0.001)
	50–65	261/23,793	10.97	3.76	3.23–4.38 (<0.001)	3.77	3.23–4.41 (<0.001)
	>65	506/15,177	33.34	11.44	10.12–12.93 (<0.001)	11.20	9.82–12.78 (<0.001)
Country of origin	Mozambican	498/105,023	4.74				
	South African	1,114/215,922	5.16	1.09	0.98–1.21 (0.121)	1.09	0.97–1.23 (0.157)
Residence status	Temporary	381/74,346	5.12				
	Permanent	1,231/246,599	4.99	0.97	0.87–1.09 (0.658)		
Time since in-migration	Non-migrant	1,098/175,266	6.26				
(years)	0–3	152/34,141	4.45	0.71	0.60–0.84 (<0.001)	0.97	0.50–1.88 (0.938)
	4–6	103/35,076	2.94	0.47	0.38–0.57 (<0.001)	0.68	0.35–1.34 (0.264)
	7–9	88/23,892	3.68	0.59	0.47–0.73 (<0.001)	0.85	0.42–1.69 (0.637)
	10–12	92/21,316	4.32	0.69	0.55–0.86 (0.001)	0.84	0.42–1.69 (0.627)
	>12	79/31,254	2.53	0.40	0.32–0.51 (<0.001)	0.39	0.19–0.78 (0.008)
Place of origin for	Non-migrant	1,098/175,266	6.26				
in-migrant	Agincourt Area	403/114,488	3.52	0.56	0.50–0.63 (<0.001)	1.16	0.59–2.25 (0.671)
	Bushbuckridge	62/17,918	3.46	0.55	0.43–0.71 (<0.001)	1.09	0.55–2.16 (0.806)
	Gauteng/Urban	40/11,584	3.45	0.55	0.40–0.76 (<0.001)	1.08	0.52–2.23 (0.839)
	Mozambique	9/1,688	5.33	0.85	0.44–1.63 (0.627)	b	b
Taxi fare (rands)	<17	368/79,296	4.64				
	17–36	810/158,155	5.12	1.10	0.97–1.26 (0.136)		
	>36	434/83,493	5.20	1.12	0.97–1.29 (0.121)		
Distance by road from the	0–5	279/63,647	4.38				
Bhubezi CHC (km)	>5	1,333/257,298	5.18	1.18	1.04–1.35 (0.013)	1.14	1.00–1.31 (0.051)
SEP quintiles	1	306/45,478	6.73				
	2	302/62,150	4.86	0.72	0.62–0.85 (<0.001)	0.79	0.67–0.93 (0.004)
	3	331/67,732	4.89	0.73	0.62–0.85 (<0.001)	0.81	0.69–0.96 (0.015)
	4	347/69,527	4.99	0.74	0.64–0.87 (<0.001)	0.88	0.74–1.03 (0.115)
	5	326/76,057	4.29	0.64	0.54–0.75 (<0.001)	0.78	0.65–0.93 (0.006)
Transport ownership	No household transport	1,245/227,473	5.47				
by household	Non-motorised transport	96/24,172	3.97	0.73	0.59–0.89 (0.002)	0.75	0.61–0.93 (0.007)
	Motorised transport (Car or Motorbike)	271/69,300	3.91	0.71	0.62–0.82 (<0.001)	0.81	0.70–0.94 (0.006)
Mean level of adult	0–5	416/60,953	6.83				
household education	6–7	443/80,363	5.51	0.81	0.71–0.92 (0.002)	0.99	0.85–1.14 (0.85)
(years)	8–9	467/94,700	4.93	0.72	0.63–0.83 (<0.001)	0.94	0.81–1.10 (0.456)
	10–11	224/63,262	3.54	0.52	0.44–0.61 (<0.001)	0.78	0.65–0.94 (0.01)
	>11	62/21,667	2.86	0.42	0.32–0.55 (<0.001)	0.79	0.58–1.06 (0.116)

a The multivariate model included variables which were showed a significant association in at least one strata in the bivariate model or were included to test *a priori* hypotheses about their significance. Time period, sex, age, country of origin time, since in-migration, place of origin, road distance from Bhubezi Community Health Centre (CHC), SEP, transport ownership and average level of household education were included.

b Strata dropped from Poisson regression due to collinearity.

There was evidence from the unadjusted and adjusted analyses that those living less than 5 km from the Bhubezi CHC and those living in households owning transport had a lower risk of dying of causes other than HIV/TB. Males had higher mortality rates than females with the effect size increasing after adjustment. There was no apparent trend for an increasing reduction in the mortality risk for each increase in SEP quintile in the adjusted or unadjusted analyses. There was evidence that increasing levels of mean adult household education led to a decrease in mortality risk; in the adjusted analysis the protective effect was only seen for those in households with greater than 10–11 years of education. For all time periods since arrival and all places of origin other than Mozambique, the unadjusted mortality rates for in-migrants were lower than those seen for non-migrants. After adjustment, a protective effect was only seen for those who had arrived more than 12 years before point of censoring. There was no evidence for a difference in unadjusted mortality rates between the temporary and permanent residents or between those of South African and Mozambican origin.

### All-cause mortality model

In a series of multivariate models with an outcome of death due to any cause, the interaction terms between covariates and the type of death (HIV/TB or other) were introduced in order to test whether the adjusted associations differed depending on the CoD (Table 4). The mortality rate ratio for males compared to females was 1.12 times higher (95% CI: 0.99–1.27) for those dying of other causes than those dying of HIV/TB. The rate ratio for South Africans compared to those of Mozambican origin was 0.85 times lower (95% CI: 0.75–0.98) for those dying of other causes compared to those dying of HIV/TB.

**Table 4:** Interaction terms for all-cause mortality model^a^

Variable	Comparison used in rate ratio	Rate ratio (other death/HIV/TB death)	95% Confidence interval (*p* value)
Sex	Male/female	1.12	0.99–1.27 (0.077)
Country of origin	South Africa/Mozambique	0.85	0.75–0.98 (0.023)
Residence status	Temporary/permanent	1.05	0.91–1.21 (0.493)
Distance from Bhubezi(km)	Greater than 5 km/5 km or less	1.02	0.86–1.20 (0.842)
SEP quintiles	Quintiles 2–5/quintile 1	0.95	0.81–1.11 (0.503)
Mean level of household education	Greater than 7 years/7 years or less	0.93	0.82–1.06 (0.294)

a Values derived from a multivariate Poisson model for all-cause mortality for deaths of known cause. The model included all terms significant at the 10% level in the bivariate model. Individual interaction terms between cause of death (HIV/TB or Other) and individual terms were introduced individually in a series of models. Results shown are for models for which convergence was achieved.

## Conclusions

In this study, we identified determinants associated with the risk of dying of HIV-related conditions and TB. HIV/TB mortality risk was lower among women, young adults (compared to older adults), those with higher levels of wealth (increased SEP), those with higher levels of education, and those of Mozambican rather than South African origin, when the effect of other factors was adjusted for in a multivariate model. Recent in-migrants had increased risk of HIV/TB death compared to non-migrants. There was evidence of a reduced risk of HIV/TB mortality among those living a short distance by road from the Bhubezi CHC and for members of households owning motorised transport. The HIV/TB mortality ratio between those households which owned motorised transport and those which did not was 1.33 times higher in 2009–2010 than in 2007–2008. There was, however, no evidence of an association between the risk of dying of HIV/TB and the public transport costs to reach the clinic from individual's home villages. The protective effects of transport ownership and education were significantly reduced in those aged less than 15 years compared to those who were older.

In the study area, there was a decrease of 27% in the HIV/TB mortality rate between 2007–2008 and 2009–2010, whilst the rate for those dying of other causes decreased by 10% over the same period. The most significant change in healthcare provision within the study site for those infected with HIV over that period was the establishment of the Bhubezi CHC. This is consistent with findings from a recent study which indicated a decrease of more than 50% in the HIV attributable mortality rate between the periods before and after the roll-out of ART at a site in KwaZulu Natal in South Africa (59). This site had a similar HIV prevalence to the site of the current study. Comparing 2007–2008 with 2009–2010 increases in the HIV/TB mortality rates were seen in certain geographical sub-regions of the study site (16) providing further evidence for the importance stated by others of identifying and responding to focussed HIV-related micro-epidemics within a more generalised epidemic (6–11).

The positive association between the distance individuals lived from a health facility and either increased rates of HIV infection or a lower take-up of ART have been reported in other studies in rural areas of sub-Saharan Africa (21, 60–62). The increased level of HIV/TB mortality for those living further than 5 km from Bhubezi CHC seen in this study is consistent with these findings. Previous studies in Uganda (23) and Malawi (22) found that increased transport costs to access treatment were associated with a lower acceptance of ART, but this was not found in this study. One reason for this difference may be that due to the relatively higher level of wealth in this setting, possibly related to the provision of social grants, the costs of transport do not constitute a significant barrier to healthcare access. Also, as we do not have data on the breakdown of the types of transport used to access the health centre and it may be that many patients use transport other than taxis.

The ownership of motorised transport was protective after controlling for SEP. It may be that ownership of transport enables easier travel for regular ART follow-up appointments. Alternatively, transport ownership may be a proxy for a household having greater monetary rather than asset wealth. Previous studies have described the financial impact on a rural African household of a member dying of HIV (63, 64). Howe et al. have discussed strengths and limitations of asset based SEP measures as opposed to income based alternatives in low and middle income countries (65). Access to greater monetary wealth would enable households to provide support for members whose earning potential is reduced due to illness. As individuals living close to the clinic would be expected to be less reliant on motorised transport to access treatment than those living further away, the finding that there was no evidence for an interaction between transport ownership and the distance individual's lived from Bhubezi CHC suggests that car ownership as a proxy for monetary wealth is a better explanation of the associations seen.

The relative difference in the risk of dying between those of Mozambican and South African origin was greater in the case of HIV/TB deaths than for those due to other causes. This may be attributable in part to different patterns of mobility between the two groups. The level of return or circular migration in this population is higher for South Africans than Mozambicans and frequent migration between areas of high and low HIV prevalence has been shown to increase the risk of HIV infection (32). A survey carried out in this area between 2011 and 2012 showed that Mozambicans had a lower prevalence of HIV infection than South Africans (44). A study on deaths from 1993 to 2011 in the site indicated no association between country of origin and all-cause mortality (24). In contrast, a study on data collected between 1992 and 2004 (39) showed increased mortality rates in the children of Mozambican immigrants. A study using data from 2001 to 2007 (40) indicated that Mozambican households had a higher proportion of household deaths than South African households. These contrasting results are likely to reflect the changing HIV epidemic dynamics over time and the on-going process of assimilation of Mozambicans into the host communities (17). It should be noted that in this area Mozambicans and South Africans share a common Shangaan heritage (17) and the cultural differences may be less between this community and others within South Africa.

A number of studies have shown that higher levels of school attendance or educational attainment are associated with a reduced risk of HIV infection (66–68). A systematic review has indicated that the association may change as the epidemic develops with higher levels of educational attainment being a risk factor at the early stages of the epidemic and protective as the epidemic develops due possibly to a greater likelihood of behavioural changes in the more educated (38). A similar time-dependent association between SEP and the risk of HIV infection has been reported, with those of higher SEP being at higher risk early in the epidemic and lower risk at a later stage (35). It has also been suggested that local inequities in wealth may be the most important factor (69). Mishra and colleagues analysed data from eight sub-Saharan African countries collected between 2003 and 2005 they found that HIV prevalence was consistently higher among those of higher SEP (70). As increased education and higher SEP were found in our study to reduce the risk of HIV/TB mortality, this suggests that this community was at a more mature stage of the epidemic. It is important to remember however, that the associations between socioeconomic determinants and the risk of becoming infected with HIV may differ from those associated with HIV mortality. There was no evidence in this study for differences in the associations of SEP or education and the mortality risk between deaths due to HIV/TB and other causes. There was also no evidence for a change in the associations over the period of this analysis.

A strength of this study is that in using data collected prospectively in the Agincourt HDSS, we were able to analyse the changing determinants of mortality over the period of time that ART was made available in this community. The inclusion of all age groups in our analysis enabled us to investigate whether associations between mortality and various risk factors were affected by age. However, the lack of CoD data for a high proportion of neonatal deaths led to exclusions from the study population which prevented us drawing strong conclusions about risk factors in this age group.

The decreases in all-cause mortality rates between the target and study populations due to the exclusions of individuals are a limitation. The overall death rate in the excluded individuals was high, 50.9 deaths/1,000 PYO; this is probably due to the fact that the lack of CoD data was an exclusion criteria. A comparison of the characteristics of those included in the study with those excluded shows that for all the categorical variables used in this analysis the distributions in the study population and excluded individuals are significantly different. This may have affected the associations with risk factors for HIV mortality derived in this analysis.

Also due to the lack of data on the clinics attended by those dying of HIV/TB and whether or not they were receiving ART, we can only make inferences about the likely impact of the opening of the Bhubezi CHC based on the overall numbers on treatment at the various clinics in the area and the times that ART became available. A programme of work to establish linkages between clinical data and the HDSS in order to better understand patterns of clinic attendance in the Agincourt study site is on-going.

The association identified between household transport ownership and decreased HIV/TB mortality is worth further investigation. An on-going research programme in this site is analysing the ways in which cash flows into individual households affect the degree of resilience those households have to stresses such as the death of a household member (64). The fact that education and SEP remained independently associated with mortality when the other factor was controlled for suggests that any programme which aims to reduce HIV incidence and mortality through structural interventions in the society needs to have a multi-pronged approach addressing both poverty alleviation and retention in education in order to maximise its effectiveness.

Despite the universal roll-out of ART in South Africa provided free at point of care, this study suggests that there remain significant barriers to accessing healthcare and subsequently remaining on treatment for particular socio-demographic and geographically defined sub-groups in the population. These factors need to be addressed if the initial success of the ART roll-out in reducing population-level mortality in South Africa is to be maintained and extended.

## Supplementary Material

Determinants of the risk of dying of HIV/AIDS in a rural South African community over the period of the decentralised roll-out of antiretroviral therapy: a longitudinal study
