# Supplementary material for: Determinants of the risk of dying of HIV/AIDS in a rural South African community over the period of the decentralised roll-out of antiretroviral therapy: a longitudinal study
Source: Glob Health Action. 2014 Nov 20;7:10.3402/gha.v7.24826. doi: 10.3402/gha.v7.24826 (PMC4245451; doi:10.3402/gha.v7.24826)
Supplement: Determinants of the risk of dying of HIV/AIDS in a rural South African community over the period of the decentralised roll-out of antiretroviral therapy: a longitudinal study [file GHA-7-24826-s001.pdf]

Supplementary materials

Supplementary Table 1: All-cause mortality rates by age for target population, study population and for deaths of known cause

	Target population [1]				Study population after exclusions [2]			
**Age Group**	Deaths /PYO	Mortality rate (Deaths/ 1000 PYO)	Mortality rate ratio	95 % confidence interval (p value)	Deaths /PYO	Mortality rate (Deaths/ 1000 PYO)	Mortality rate ratio	95 % confidence interval (p value)
**0 - 4**	511/40471	12.63			389/39035	9.97		
**5 - 14**	105/75403	1.39	0.10	0.08 - 0.13 (<0.001)	77/72418	1.06	0.10	0.08 - 0.13 (<0.001)
**15 - 24**	183/80375	2.28	0.16	0.14 - 0.19 (<0.001)	133/76946	1.73	0.16	0.13 - 0.19 (<0.001)
**25 - 34**	628/54651	11.49	0.81	0.72 - 0.91 (<0.001)	487/51759	9.41	0.84	0.73 - 0.96 (0.012)
**35 - 44**	630/34210	18.42	1.31	1.16 - 1.47 (<0.001)	507/32429	15.63	1.40	1.23 - 1.61 (<0.001)
**45 - 54**	440/21486	20.48	1.44	1.25 - 1.65 (<0.001)	354/20589	17.19	1.53	1.31 - 1.79 (<0.001)
**55 - 64**	343/13003	26.38	1.85	1.60 - 2.13 (<0.001)	277/12591	22.00	1.95	1.67 - 2.29 (<0.001)
**>= 65**	785/15791	49.71	3.25	2.89 - 3.65 (<0.001)	666/15177	43.88	3.63	3.19 - 4.13 (<0.001)
**total**	**3625/335392**	**10.81**			**2890/320945**	**9.00**		

[1] Includes only those individuals resident at the midpoint of the analysis 1st Jan 2009
[2] Individuals with death of unknown cause or with data missing for covariates excluded

Supplementary Table 2: Breakdown of the causes for deaths occurring between 2007 and 2010 in the study population as assigned by the InterVA-4 model

Cause of Death [1,2]	Total	%
HIV/AIDS	724	25.05
Pulmonary tuberculosis	554	19.17
Acute respiratory infection including pneumonia	490	16.96
Neoplasms	178	6.16
Respiratory disorders	142	4.91
Other Infectious and Parasitic Diseases	178	6.16
Other diseases of the circulatory system	122	4.22
Stroke	115	3.98
Other external causes	92	3.18
Road Traffic Accident	67	2.32
Gastrointestinal disorders	65	2.25
Diabetes	49	1.70
Other non-communicable	24	0.83
Other nutritional and endocrine disorders	17	0.59
Neonatal death	55	1.90
Pregnancy related	7	0.24
Epilepsy	7	0.24
Renal failure	4	0.14
	**2890**	

[1] WHO 2012 cause of death categories as defined in WHO (2012) Verbal autopsy standards: the 2012 WHO verbal autopsy instrument http://www.who.int/healthinfo/statistics/WHO_VA_2012_RC1_Instrument.pdf.

[2] The table contains the cause of death for all deaths for which the InterVA-4 model was able to assign a cause, there were 569 additional deaths for which no cause could be assigned over the period of analysis.

Supplementary Table 3: Comparison of individual education and average adult education as predictors of the risk of dying of HIV/TB for those aged over 18 years in the study population

Variable	Category	Deaths/PYO [1]	Mortality rate [illegible]	Rate Ratio	95 % confidence interval (p value)
Mean level of household education	0 – 5	325/38978	8.33		
	6 – 7	336/52263	6.43	0.77	0.66 – 0.90 (0.001)
	8 – 9	343/64002	5.36	0.64	0.55 – 0.75 (<0.001)
	10 – 11	161/44124	3.65	0.44	0.36 – 0.53 (<0.001)
	> 11	42/14741	2.85	0.34	0.25 – 0.47 (<0.001)
Individual level of education	0 – 5	459/75114	6.28		
	6 – 7	156/19292	8.09	1.29	1.07 – 1.54 (0.006)
	8 – 9	136/22401	6.07	0.97	0.80 – 1.17 (0.727)
	10 – 11	167/35249	4.74	0.75	0.63 – 0.90 (0.002)
	> 11	241/61773	3.90	0.62	0.53 – 0.73 (<0.001)

[1] PYO = Person years of observation

Supplementary Table 4: Interaction terms with Time period for AIDS/TB mortality model 1

Variable	Comparison used in Rate Ratio	Rate Ratio (2009 – 2010 / 2007 – 2008)	95 % confidence interval (p value)
**SEP level**	(Quintiles 2 – 5 /Quintile 1)	0.91	0.70 – 1.18 (0.479)
**Mean level of household education**	Greater than 7 years / 7 years or less	0.97	0.77 – 1.21 (0.762)
**Household Transport ownership**	Transport owned / No household transport	1.33	1.00 – 1.77 (0.054)
**Country of origin**	South Africa /Mozambique	0.97	0.76 – 1.24 (0.807)
**Distance by road to Bhubezi Community Health Centre**	Greater than 5 km / 5 km or less	0.97	0.72 – 1.31 (0.854)

[1] Values derived from a multivariate poisson model for all-cause mortality for AIDS/TB mortality. The model included all terms significant at the 10 % level in the bivariate model. Individual interaction terms between Time period (2009 – 2010 vs 2007 - 2008) and individual variables were introduced individually in a series of models.

Supplementary Table 5: Interaction terms with Age group for the AIDS/TB mortality model 1

Variable	Comparison used in Rate Ratio	Rate Ratio (Less than 15 years / 15 years and older)	95 % confidence interval (p value)
**SEP level**	(Quintiles 2 – 5 /Quintile 1)	1.35	0.85 – 2.16 (0.210)
**Mean level of household education**	Greater than 7 years / 7 years or less	0.70	0.47 – 1.05 (0.085)
**Household Transport ownership**	Transport owned / No household transport	0.60	0.34 – 1.06 (0.077)
**Country of origin**	South Africa /Mozambique	0.75	0.51 – 1.12 (0.157)
**Gender**	Male / Female	0.92	0.63 – 1.33 (0.661)

[1] Values derived from a multivariate poisson model for all-cause mortality for AIDS/TB mortality. The model included all terms significant at the 10 % level in the bivariate model. Individual interaction terms between Time period (2009 – 2010 vs 2007 - 2008) and individual variables were introduced individually in a series of models.

Supplementary Table 6: Components of the asset index.

**Household assets**	**Asset Category**
Structure completion status	Dwelling
Plans to extend structure	Dwelling
Construction material – roof	Dwelling
Construction material – walls	Dwelling
Construction material – floor	Dwelling
Total number of bedrooms	Dwelling
Separate kitchen	Dwelling
Separate living/dining room	Dwelling
Toilet location	Sanitation
Toilet type	Sanitation
Mains water supply	Sanitation
Availability of water supply	Sanitation
Primary power source for lights and appliances	Power
Primary power source for cooking	Power
Functioning stove	Modern Assets
Functioning refrigerator	Modern Assets
Functioning TV/hi-fi/stereo	Modern Assets
Functioning video machine/DVD player	Modern Assets
Functioning satellite dish	Modern Assets
Functioning radio	Modern Assets
Functioning landline telephone	Modern Assets
Functioning cell telephone	Modern Assets
Functioning car or truck	Modern Assets
Functioning motor bike	Modern Assets
Functioning bicycle	Modern Assets
Functioning animal drawn cart	Livestock
Number of cattle owned	Livestock
Number of goats owned	Livestock
Number of chickens owned	Livestock
Number of pigs owned	Livestock

Supplementary Table 7: Comparison of the age of death between those included and excluded from the study population

Variable	Category	Study Population N (%)	Excluded Individuals N (%)	Target Population N (%)	p –value *
Age of Death	< 1	246 (8.5)	83 (11.3)	329 (9.1)	0.001
	1 – 4	143 (5.0)	39 (5.3)	182 (5.0)	
	5 – 14	77 (2.7)	28 (3.8)	105 (2.9)	
	5 - 14	1311 (45.4)	357 (48.6)	1668 (46.0)	
	50 - 65	475 (16.4)	111 (15.1)	586 (16.2)	
	> 65	638 (22.1)	117 (15.9)	755 (20.8)	
	**TOTAL**	**2890**	**735**	**3625**	

* p values for the chi-squared test comparing those included in the study population with those who were excluded.

Supplementary Table 8: Comparison of the mean value of continuous variables in the target and study populations

Variable	Mean value in study population	Mean value in excluded individuals	Mean value in target population	p-value *
**Age at mid point in the study (years)**	24.74	25.77	24.70	< 0.001
**Mean household socio-economic status**	2.56	2.52	2.56	< 0.001
**Mean household average adult education (years)**	7.71	7.67	7.70	0.224

* Tested using a two sample t-test, p-value equals the probability that the difference or a larger difference between the mean values of the excluded individuals and the study populations would arise by chance if there was no true difference.

Supplementary Table 8: Comparison of the distribution of categorical variables in the target and study populations.

Variable	Category	Study Population N (%)	Excluded Individuals N (%)	Target Population N (%)	p –value *
Gender	Female	55938 (53.2)	4400 (54.3)	60338 (53.3)	0.051
	Male	49211 (46.8)	3700 (45.7)	52911 (46.7)	
Age at Mid point	< 5	11577 (12.4)	374 (9.4)	11951 (12.3)	< 0.001
	15 - 49	21213 (22.8)	834 (20.9)	22047 (22.7)	
	5 - 14	50403 (54.1)	2412 (60.4)	52815 (54.4)	
	50 - 65	6094 (6.5)	210 (5.3)	6304 (6.5)	
	> 65	3836 (4.1)	162 (4.1)	3998 (4.1)	
Country of origin	Mozambican	34337 (32.7)	2748 (35.0)	37085 (32.8)	< 0.001
	South African	70812 (67.3)	5115 (65.0)	75927 (67.2)	
Residence status	Temporary	24183 (23.0)	1896 (25.2)	26079 (23.2)	< 0.001
	Permanent	80966 (77.0)	5617 (74.8)	86583 (76.9)	
Time since in-migration (years)	Non-Migrant	55399 (52.7)	750 (9.25)	56149 (49.6)	< 0.001
	0 - 3	18831 (17.9)	5340 (65.9)	24171 (21.3)	
	4 – 6	10042 (9.6)	760 (9.4)	10802 (9.5)	
	7 – 9	6794 (6.5)	494 (6.1)	7288 (6.4)	
	10 – 12	5822 (5.5)	224 (2.8)	6046 (5.3)	
	> 12	8261 (7.9)	536 (6.6)	8797 (7.8)	
Place of origin for in-migrant	Non-Migrant	55399 (52.7)	750 (13.8)	56149 (50.8)	< 0.001
	Agincourt Area	37605 (35.8)	3660 (67.3)	41265 (37.3)	
	Bushbuckridge	6992 (6.7)	566 (10.4)	7558 (6.8)	
	Gauteng /Urban	4417 (4.2)	349 (6.4)	4766 (4.3)	
	Mozambique	736 (0.7)	113 (2.1)	849 (0.8)	
Taxi fare (Rands)	< 17	25178 (24.0)	2186 (27.0)	27364 (24.2)	< 0.001
	17 - 36	51737 (49.2)	3647 (45.0)	55384 (48.9)	
	> 36	28234 (26.9)	2271 (28.0)	30505 (26.9)	
Distance by road from the Bhubezi community helath centre (km)	0 - 5	20177 (19.2)	1886 (23.3)	22063 (19.5)	< 0.001
	> 5	84972 (80.8)	6218 (76.7)	91190 (80.5)	
SEP quintiles	1	16194 (15.4)	661 (18.2)	16855 (15.5)	< 0.001
	2	20566 (19.6)	747 (20.5)	21313 (19.6)	
	3	22201 (21.1)	728 (20.0)	22929 (21.1)	
	4	22353 (21.2)	762 (21.0)	23115 (21.3)	
	5	23835 (22.7)	738 (20.3)	24573 (22.6)	
Transport ownership by household	No household transport	76223 (72.5)	7196 (88.8)	83419 (73.7)	< 0.001
	Non-motorised transport	7583 (7.2)	196 (2.4)	7779 (6.87)	
	Motorised transport (Car or Motorbike)	21343 (20.3)	712 (8.8)	22055 (19.5)	
Mean level of adult household education (years)	0 – 5	20486 (19.5)	1899 (23.4)	22385 (19.8)	< 0.001
	6 – 7	26465 (25.2)	1594 (19.7)	28059 (24.8)	
	8 – 9	30889 (29.4)	1801 (22.2)	32690 (28.9)	
	10 – 11	20465 (19.5)	1543 (19.0)	22008 (19.4)	
	> 11	6844 (6.5)	1267 (15.6)	8111 (7.2)	

* p values for the chi-squared test comparing those included in the study population with those who were excluded.

Supplementary Table 9: Comparison of the age of death between those included and excluded from the study population

Variable	Category	Study Population N (%)	Excluded Individuals N (%)	Target Population N (%)	Chi-squared p –value *
Age of Death	< 1	246 (8.5)	83 (11.3)	329 (9.1)	0.001
	1 – 4	143 (5.0)	39 (5.3)	182 (5.0)	
	5 – 14	77 (2.7)	28 (3.8)	105 (2.9)	
	5 - 14	1311 (45.4)	357 (48.6)	1668 (46.0)	
	50 - 65	475 (16.4)	111 (15.1)	586 (16.2)	
	> 65	638 (22.1)	117 (15.9)	755 (20.8)	
	**TOTAL**	**2890**	**735**	**3625**	
